# Association between the −794 (CATT)_5–8_ *MIF* Gene Polymorphism and Susceptibility to Acute Coronary Syndrome in a Western Mexican Population

**DOI:** 10.1155/2014/704854

**Published:** 2014-07-03

**Authors:** Emmanuel Valdés-Alvarado, José Francisco Muñoz-Valle, Yeminia Valle, Elena Sandoval-Pinto, Ilian Janet García-González, Angélica Valdez-Haro, Ulises De la Cruz-Mosso, Héctor Enrique Flores-Salinas, Jorgé Ramón Padilla-Gutiérrez

**Affiliations:** ^1^Instituto de Investigación en Ciencias Biomédicas, Centro Universitario de Ciencias de la Salud, Universidad de Guadalajara, Sierra Mojada 950, Edificio Q, Primer Piso, Colonia Independencia, 44350 Guadalajara, JAL, Mexico; ^2^Doctorado en Ciencias Biomédicas, Universidad de Guadalajara, Sierra Mojada 950, Colonia Independencia, 44350 Guadalajara, JAL, Mexico; ^3^Doctorado en Genética Humana, Universidad de Guadalajara, Sierra Mojada 950, Colonia Independencia, 44350 Guadalajara, JAL, Mexico; ^4^IMSS, Centro Medico Nacional de Occidente, Belisario Dominguez 1000, Colonia Independencia, 44340 Guadalajara, JAL, Mexico

## Abstract

The macrophage migration inhibitory factor (*MIF*) is related to the progression of atherosclerosis, which, in turn, is a key factor in the development of acute coronary syndrome (ACS). *MIF* has a CATT short tandem repeat (STR) at position −794 that might be involved in its expression rate. The aim of this study was to investigate the association between the −794 (CATT)_5–8_ *MIF* gene polymorphism and susceptibility to ACS in a western Mexican population. This research included 200 ACS patients classified according to the criteria of the American College of Cardiology (ACC) and 200 healthy subjects (HS). The −794 (CATT)_5–8_ *MIF* gene polymorphism was analyzed using a conventional polymerase chain reaction (PCR) technique. The 6 allele was the most frequent in both groups (ACS: 54% and HS: 57%). The most common genotypes in ACS patients and HS were 6/7 and 6/6, respectively, and a significant association was found between the 6/7 genotype and susceptibility to ACS (68% versus 47% in ACS and HS, resp., *P* = 0.03). We conclude that the 6/7 genotype of the *MIF* −794 (CATT)_5–8_ polymorphism is associated with susceptibility to ACS in a western Mexican population.

## 1. Introduction

Acute coronary syndrome (ACS) is characterized by acute, regional reductions in coronary blood flow and myocardial ischemia [1]. ACS describes a spectrum of clinical manifestations, including unstable angina (UA), non-ST-segment elevation myocardial infarction (NSTEMI), and ST-segment elevation myocardial infarction (STEMI) [1–3]. The vast majority of ACS is triggered by disruption of an atherosclerotic plaque [1]. Atherosclerosis is a chronic inflammatory disease of the arterial wall, characterized by endothelial dysfunction, intimal hyperplasia, and smooth muscle proliferation, as well as deposition of lipids and formation of microvessels within the vascular wall [1, 4–6]. Endothelial dysfunction is accompanied by the expression of adhesion molecules (such as the vascular cell adhesion molecule- (VCAM-) 1) [7, 8] and chemokines [4, 9]. The recruitment of inflammatory cells is triggered by the production of cytokines (such as IL-1*β*, IL-6, IL-8, TNF-*α*, and CCL2) [6] within the plaque microenvironment. Chemokines are released from endothelial cells, mast cells, platelets, macrophages, and lymphocytes [10].

The macrophage migration inhibitory factor (*MIF*) is a molecule that consists of 115 amino acids [11]; it was described as the main cytokine involved in attracting immune cells such as macrophages and T and B cells [12, 13]. The secretion of* MIF* by inflammatory cells can be induced by exposure to oxidized low-density lipoprotein or other cytokines, such as TNF-*α* and interleukin-c [13, 14].* MIF* activates the expression of various proinflammatory cytokines and chemokines and recruits macrophages to the site of atherosclerosis [12].* MIF* participates in the pathogenesis of inflammatory and atherosclerosis processes [12, 13, 15–17].

The* MIF* gene is located at 22q11.2 [18] and contains several polymorphisms, including the rs5844572. This genetic marker is a CATT short tandem repeat (STR) at position −794, with five to eight length variants (alleles 5 to 8). There is an association between the length of the repeats and the expression of the genetic marker; the higher alleles (CATT6, CATT7, and CATT8) show higher expression of the gene [13]. Since* MIF* is an inflammatory mediator and given the role of genetic factors that modify its expression,* MIF* could contribute to atherosclerosis and susceptibility to ACS. The aim of this study was to investigate the association between the −794 (CATT)_5–8_ *MIF* gene polymorphism and susceptibility to ACS in a western Mexican population.

## 2. Materials and Methods

### 2.1. Subjects

The study group included 200 ACS unrelated patients recruited from Hospital de Especialidades del Centro Médico Nacional de Occidente del Instituto Mexicano del Seguro Social (CMNO-IMSS) and classified according to the criteria of the American College of Cardiology (ACC) [19]. As a control group, 200 unrelated healthy subjects (HS) were recruited from the general population of western Mexico. We applied a standardized questionnaire to the HS group and applied routine laboratorial clinical assessments to detect any potential alterations, and those subjects with any clinical alterations were excluded of the study. We considered Mexican Mestizo subjects, only those individuals who for three generations, including their own, had been born in western Mexico. The participation of the subjects was voluntary and all signed a written informed consent. The study conforms to the ethical principles contained in the declaration of Helsinki, and ethical approval was obtained from Centro Universitario de Ciencias de la Salud, CUCS, UdeG (C.I. 069-2012).

### 2.2. Genotyping of the* MIF* −794 (CATT)_5–8_

Genomic DNA was extracted from peripheral blood leukocytes using Miller's Technique [20]. Genotyping of the STR −794 (CATT)_5–8_ polymorphism was achieved by conventional PCR and polyacrylamide gel electrophoresis using the primers reported by Radskate et al. [21]. Cycling conditions were as follows: an initial denaturation at 95°C for 4 min followed by 30 cycles of 30s at 95°C, 30s at 60°C, and 30s at 72°C and then a final extension of 2 min at 72°C. Amplification products were further electrophoresed on a 19: 1 10% polyacrylamide gel. Allele identification was done by using a 10-bp (Invitrogen) and homemade allelic ladders containing pooled samples. In addition, as genotyping control, automatized sequencing of one random sample of each homozygote genotype allowed confirmation of results (ABI PRISM 377 Genetic Analyzer, Applied Biosystems, Foster City, CA, USA).

### 2.3. Statistical Analysis

The Hardy-Weinberg equilibrium test and genotype and allele frequencies were calculated by the chi-square test or Fisher's exact test, when applicable. Odds ratios (OR) and 95% confidence intervals (95% CI) were calculated to test the probability that the genotype and allele frequencies were associated with ACS. A *P* value <0.05 was considered to be statistically significant. All the statistical analyses were done with the Stata 9.0 software.

In this study the sample size was calculated according to the minor allele frequency of the −794 (CATT)_5–8_ *MIF* gene polymorphism reported in Mexican Mestizo population by Llamas-Covarrubias et al. [16] and we obtained at least 164 alleles (22.14%) using the Kelsey formula. It means that in order to detect differences, we needed at least 82 individuals each group with 95% confidence interval and statistical power of 80%. In this respect 200 individuals were included exceeding by far the required sample size.

## 3. Results

### 3.1. Clinical Characteristics

All clinical characteristics are shown in Table 1. The median age of HS and ACS groups was 61 and 63 years, respectively. The gender distribution among ACS individuals was 76% male and 24% female. The most prevalent clinical diagnosis in the ACS group was STEMI (76%), while obesity was the most common risk factor (61%), followed by high blood pressure (HBP: 60%). Also, in the ACS group other clinical parameters such as troponin I (TnI), troponin T (TnT), creatine phosphokinase (CPK), and creatine kinase (CK) were evaluated in order to find a possible genetic association. However, we did not find any statistical association with −794 (CATT)_5–8_ *MIF* gene polymorphism.

### 3.2. Distribution of the* MIF* −794 (CATT)_5–8_ Polymorphism

No deviation from the Hardy-Weinberg equilibrium was detected in the −794 (CATT)_5–8_ *MIF* polymorphism (*P* = 0.16). The allele and genotype frequencies in both the ACS and HS groups are shown in Table 2. Allele 6 was the most frequent in both groups (ACS: 54% and HS: 57%). The most common genotypes in ACS and HS subjects were 6/7 and 6/6, respectively. A significant association was found between the 6/7 genotype and susceptibility to ACS (68% versus 47% in ACS and HS, resp., *P* = 0.03).

### 3.3. Risk Factors in the ACS Group Related to the Genotypes of the −794 (CATT)_5–8_ *MIF* Polymorphism

We also stratified the main risk factors for development of ACS by genotype (Table 3). Despite no significant differences, we observed that the 6/7 genotype was the most frequent in each subgroup and that the 5/5 genotype was the less common.

## 4. Discussion

Acute coronary syndrome is a multifactorial disease arising through a combination of both environmental and genetic risk factors. Several studies have reported a relationship between atherosclerosis and ACS [8, 22]. Inflammation regulates the stability of the atherosclerotic plaque [8]. Activated endothelial cells express adhesion molecules and favor the recruitment of monocytes to the endothelium. These in turn release proinflammatory cytokines including IL-1, IL-6, TNF-*α*, and* MIF*. Several researches have focused on the role of* MIF* in the atherosclerosis process. Ayoub et al. report that* MIF* enhances macrophage uptake of oxidized LDL in the progression of atherosclerosis [23]. Zernecke et al. reported a correlation between the expression of* MIF* and an increased intima-media thickness and also with lipid deposition in carotid artery plaques [4]; White et al. reported a proinflammatory role for* MIF* in acute myocardial infarction [24]; Müller et al. demonstrated that the expression of* MIF* is significantly higher in ACS patients [15].

The* MIF* promoter has a CATT STR polymorphism at position −794. This genetic marker has five to eight variants (CATT)_5–8_. A higher repeat number is associated with an increase in the gene expression [13]. In the present research, we reviewed the association of the −794 (CATT)_5–8_ *MIF* polymorphism with ACS in Mexican Mestizo individuals. The genotype and allele frequencies were distributed in a similar way to a previous study with a Mexican Mestizo population by Llamas-Covarrubias et al. [16]. We also compared the frequencies with those of Caucasian American and African American populations [25] and observed that the genotype frequencies in our population differed from them; they found that the genotype 6/6 was the most frequent, while the 5/8 and 6/8 genotypes were less frequent in Caucasian Americans and African Americans, respectively. In addition, a statistical difference was found in genotypic and allelic distributions when compared with an African ethnic group where the 5/6 genotype and the 5 allele showed the highest frequency [26]; these differences could be attributed to the ancestry of the population. The Mexican Mestizo population is a crossbreed of Amerindian, European, and African populations, with an estimated contribution of 21–25%, 60–64%, and 15%, respectively (Rubi-Castellanos, 2009).

In this study, we found an association between the 6/7 genotype of the* MIF* −794 (CATT)_5–8_ polymorphism and susceptibility to ACS in a western Mexican population. The 6/7 carriers present 1.77 more susceptibility to develop ACS. We did not find any association with respect to the allelic frequencies. There is also another single nucleotide polymorphism in the promoter of the* MIF* gene at the position −173 that has been previously analyzed and related to the inflammatory response in coronary bypass surgery [27] and coronary alterations in children with Kawasaki disease [28].

Recent studies support the fact that* MIF* is a pleiotropic cytokine mainly released from macrophages that has been shown to be increasingly expressed during atherogenesis, oxLDL being a major inducer [14, 23, 24]. ACS is a pathology where inflammation favors the recruitment of monocytes to the atherosclerotic plaque; otherwise, new studies have described the functional effect of* MIF* −794 (CATT)_5–8_, showing that the CATT_5_ allele has the lowest transcriptional activity and the CATT_6–8_ alleles increase the expression rate. This evidence validates the association found between the 6/7 genotype of the* MIF* −794 (CATT)_5–8_ polymorphism and susceptibility to ACS. The molecular mechanisms that regulate* MIF* expression have been poorly studied. Recently, Chen et al. showed that the transcriptional repressor HBP1 (HMG box-containing protein 1) negatively regulates* MIF* expression, but this is still under investigation [29].

We also stratified the risk factors in the ACS group regarding the genotypes of the −794 (CATT)_5–8_ *MIF* polymorphism. As we mentioned,* MIF* has an important function in inflammatory processes (Kleemann et al. [30]), suggesting that* MIF* controls the development of metabolic pathologies associated with ACS risk factors such as obesity and type 2 diabetes mellitus. Nevertheless, we did not find any association between the genotypes of the −794 (CATT)_5–8_ *MIF* polymorphism and the risk factors in the ACS group. However a trend is shown between the 6/7 genotype and obesity, type 2 diabetes mellitus, dyslipidemia, and high blood pressure. We want to highlight that, as a limitation of the study, the sample size in each subgroup is not enough to find an association, but there is a marked tendency with the higher alleles, which are responsible for a higher expression.

It is worth noting that the −794 (CATT)_5–8_ promoter polymorphism of* MIF* has not been studied in relation to susceptibility to ACS.

## 5. Conclusion

The 6/7 genotype of the* MIF* −794 (CATT)_5–8_ polymorphism is related with susceptibility to ACS in a western Mexican population.

## Figures and Tables

**Table 1:** Clinical characteristics in the study population.

Parameter	ACS *n* (%)	HS *n* (%)	
Age(years)	63 (37–91)∗	61 (26–91)∗	
Gender			
Male	152 (76)	136 (68)	
Female	48 (24)	64 (32)	
ACS diagnosis		ACS risk factor *n* (%)	
UA	26 (13)	Obesity DM2 DYS HBP	79 (61) 50 (50) 93 (47) 119 (60)
NSTEMI	22 (11)		
STEMI	152 (76)		

Non-ST-segment elevation myocardial infarction (NSTEMI), unstable angina (UA), and ST-segment elevation myocardial infarction (STEMI). DM2: type 2 diabetes mellitus, DYS: dyslipidemia HBP: high blood pressure. ∗Minimum–maximum.

**Table 2:** Allele and genotype distributions of −794 (CATT)_5–8_ *MIF* polymorphisms in ACS and HS.

Polymorphism	ACS *n* = 200 (%)	HS *n* = 200 (%)	OR (CI 95%); P*
−794 CATT_5–8_ * MIF *			
Genotype			
5,5	9 (4)	6 (3)	2.76 (0.71–12.86); 0.09
5,6	41 (21)	52 (26)	0.93 (0.52–1.66); 0.79
5,7	19 (9)	18 (9)	1.45 (0.64–3.35); 0.33
6,6^§^	53 (27)	65 (33)	1
6,7	**68 (34)**	**47 (23)**	**1.77 (1.05–2.98); 0.03**
7,7	10 (5)	12 (6)	1.02 (0.36–2.81); 0.96
Allele			
5	78 (19)	82 (20)	1.01 (0.70–1.45); 0.94
6^§^	215 (54)	229 (57)	1
7	107 (27)	89 (23)	1.28 (0.91–1.79); 0.15

ACS: acute coronary syndrome, HS: healthy subjects, OR: odd ratio, and CI: confidence interval. ∗*P* < 0.05.

*MIF*: macrophage migration inhibitory factor.

**Table 3:** Risk factors in the ACS group related to the genotypes of the −794 (CATT)_5–8_ *MIF* polymorphism.

Phenotype	Genotypes of the −794 (CATT)_5–8_ * MIF* polymorphism								
	ACS (*n*)	5,5	5,6	5,7	6,6	6,7	7,7	OR (CI 95%)	*P*
Obesity	80	4 (5.06)	15 (18.99)	6 (7.59)	22 (27.85)	25 (31.65)	7 (8.86)	3.29 (0.65–21.48)	0.10
DM2	100	3 (3)	20 (20)	10 (10)	27 (27)	34 (34)	6 (6)	0.48 (0.07–2.57)	0.33
DYS	93	5 (5.38)	22 (23.76)	6 (6.45)	24 (25.81)	30 (32.26)	6 (6.45)	0.56 (0.15–1.89)	0.30
HBP	119	6 (5.04)	26 (21.85)	9 (7.56)	32 (26.89)	37 (31.09)	9 (7.56)	5.91 (0.71–270.94)	0.07

DM2: type 2 diabetes mellitus, DYS: dyslipidemia, and HBP: high blood pressure.
